# Serum protein electrophoretic pattern in piglets during the early postnatal period

**DOI:** 10.1038/s41598-021-96957-6

**Published:** 2021-09-02

**Authors:** Csilla Tóthová, Robert Link, Petronela Kyzeková, Oskar Nagy

**Affiliations:** 1 Clinic of Ruminants, University of Veterinary Medicine and Pharmacy, Komenského 73, 041 81 Košice, Slovak Republic; 2 Clinic of Swine, University of Veterinary Medicine and Pharmacy, Komenského 73, 041 01 Košice, Slovak Republic

**Keywords:** Physiology, Biomarkers

## Abstract

The pattern of serum proteins, the typical features of the electrophoretogram in newborn piglets and during their postnatal development is not completely described. Therefore, the aim of this study was to characterize the changes in serum protein electrophoretic pattern and features of the electrophoretograms during the early postnatal period. Significant changes during the monitored period were found in all evaluated parameters (*P* < 0.001). The most marked changes were observed mainly in the period before weaning. The concentrations of total proteins, albumin and γ-globulins were before colostrum intake low, γ-globulins represented the smallest proportion of protein fractions. The proportion of α_1_-globulins was after birth a dominant protein fraction. Significant increase of total proteins, α_2_-, β- and γ-globulins and decrease of α_1_-globulins was found 2 days after colostrum intake. The albumin and A/G values increased after birth gradually until weaning. After weaning a significant changes were found in absolute concentrations of total protein and albumin, and in relative values of β-globulin fractions. Presented results showed marked developmental alterations in the serum protein pattern in piglets along with the age. The study also brings new knowledge in the field of description of typical features of electrophoretograms in the observed period of piglet’s life.

## Introduction

At birth, the piglets are physiologically not mature and their growth involves several morphological, metabolic and physiological changes, which may be reflected also by biochemical changes. Glucogenesis, lipogenesis, as well as the liver functions are not fully developed, which is related also to limited protein synthesis^1,2^. Thus, the concentrations of serum proteins in piglets during most of the fetal period and immediately after birth are markedly lower compared to those in adult pigs, and the measured values are only around 20 g/l^3,4^. The main components of serum proteins in newborn piglets are glycoproteins (especially α_1_-acid glycoprotein, fetuin, α_1_-antitrypsin, α_1_-fetoprotein), while albumin is only a minor component (in contrast to nearly 60% of total serum proteins in newborn calves) and immunoglobulins are practically absent^5–8^. The placenta of sows is classified as epitheliochorial type, which does not allow maternal immunoglobulins to be transferred to the fetal circulation. Therefore, the survival of piglets and their sufficient growth and development depend on the intake of colostrum, which provides them not only with energy for growth and heat production, but also with passive immunity due to the intestinal uptake of immunoglobulins^9,10^. Colostrum is the ‘first milk’ and an essential source of energy, nutrients and is critical for development of the piglets’ immune system and optimum lifetime performance. Providing satisfactory colostrum intake to piglets is a major challenge. Because piglets are born with little energy and very few protecting antibodies, colostrum intake is the main determinant of their survival through provision of energy and immune protection and has potential long-term effects. As the transfer of macromolecules across the intestinal mucosa is possible only for a short time after birth, the period of colostrum intake is the most important^11^. During this period the piglets obtain the immune defenses necessary for a sustained growth and development^12^. Weaning and the immediate post-weaning period are critical stressful life periods for piglets, when they need to be adapted to the separation from the sow and to receive complex solid feed. At this time, piglets are very susceptible to gastrointestinal disorders and respiratory infections.

The parameters related to protein metabolism belong to relevant markers, which may reflect the changes in the organism associated with growth and development, as well as changes in nutrition. During the postnatal development, especially in the first days of life, the serum protein pattern of piglets may undergo profound changes, the importance of which is not yet completely understood. Some studies performed to evaluate the developmental changes in the serum proteins were conducted many years ago, and were oriented predominantly to the evaluation of separate serum proteins, especially acute phase proteins, not the complete serum protein pattern^6,13,14^. The findings of these studies showed that in porcine fetuses, fetuin, α-fetoprotein and α1-antitrypsin were the major serum proteins, but albumin was not detected. The serum concentrations of α-fetoprotein, α1-antitrypsin and transferrin fell progressively till birth, whereas those of fetuin, albumin and α1-glycoprotein started to increase. At birth, α1-glycoprotein and fetuin were present in very high concentrations, but albumin still represented only about 7% of serum proteins. Serum protein electrophoresis has been successfully applied in clinical laboratories for the separation and analysis of blood serum proteins, as well as to detect protein abnormalities^15,16^. However, there are no published papers available describing the electrophoretogram in piglets to show the typical features, nature and type of protein zones in this animal species. Since many physiological and functional changes occur in piglets during intensive growth and maturation in the neonatal period, we expected that these processes may lead to changes also in the composition of serum proteins. Therefore, the objective of this study was to characterize the neonatal piglet serum protein electrophoretic pattern, and to evaluate the changes in the concentrations of main serum protein fractions before and after natural sucking and during the postnatal development including the period from immediately after birth (pre-colostral) to period after weaning. The aim of the study was also to describe the typical features of the electrophoretograms of piglets during this evaluated period.

## Results

**Figure 1:**
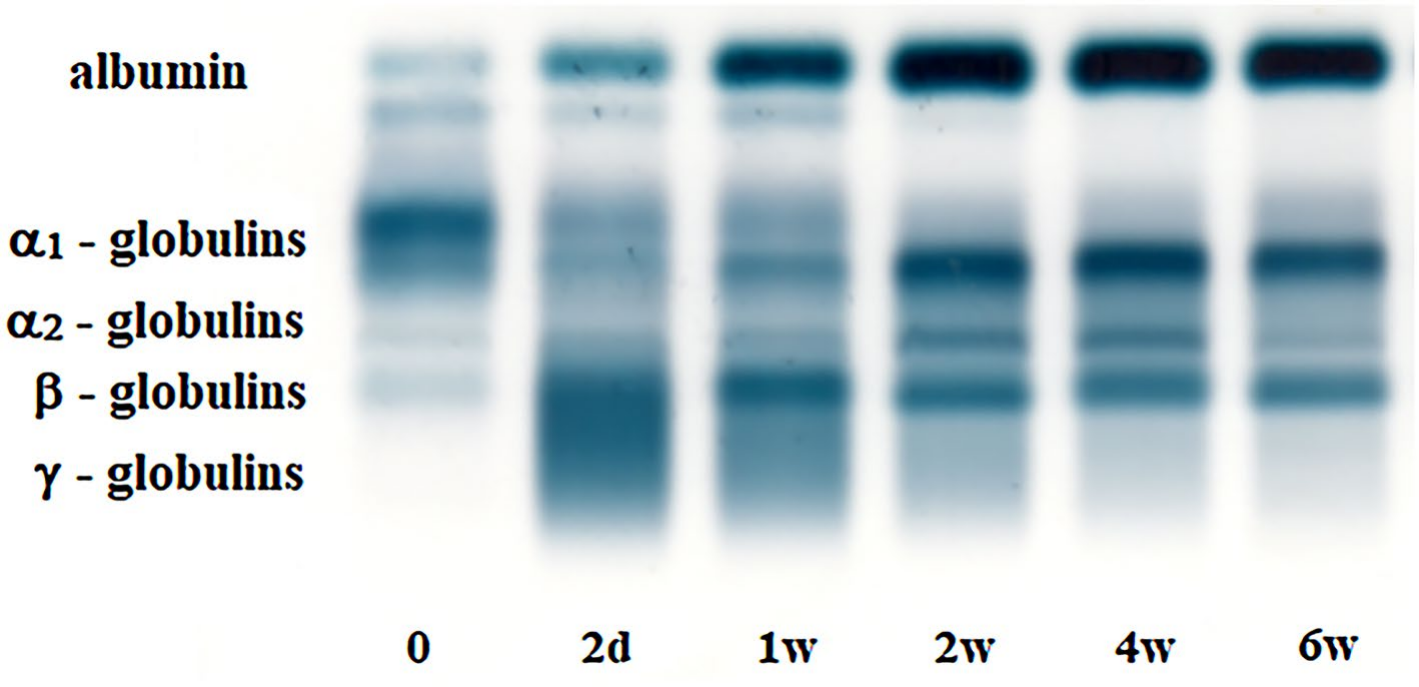
Electrophoretograms showing the protein fractions and changes in their proportion in a piglet during the early postnatal period—before colostrum inteke (0), on day 2 (2d), 1 week (1w), 2 weeks (2w), 4 weeks (4w) and 6 weeks (6w) after birth. (The display of the original gel is included in a Supplementary Information file).

**Figure 2:**
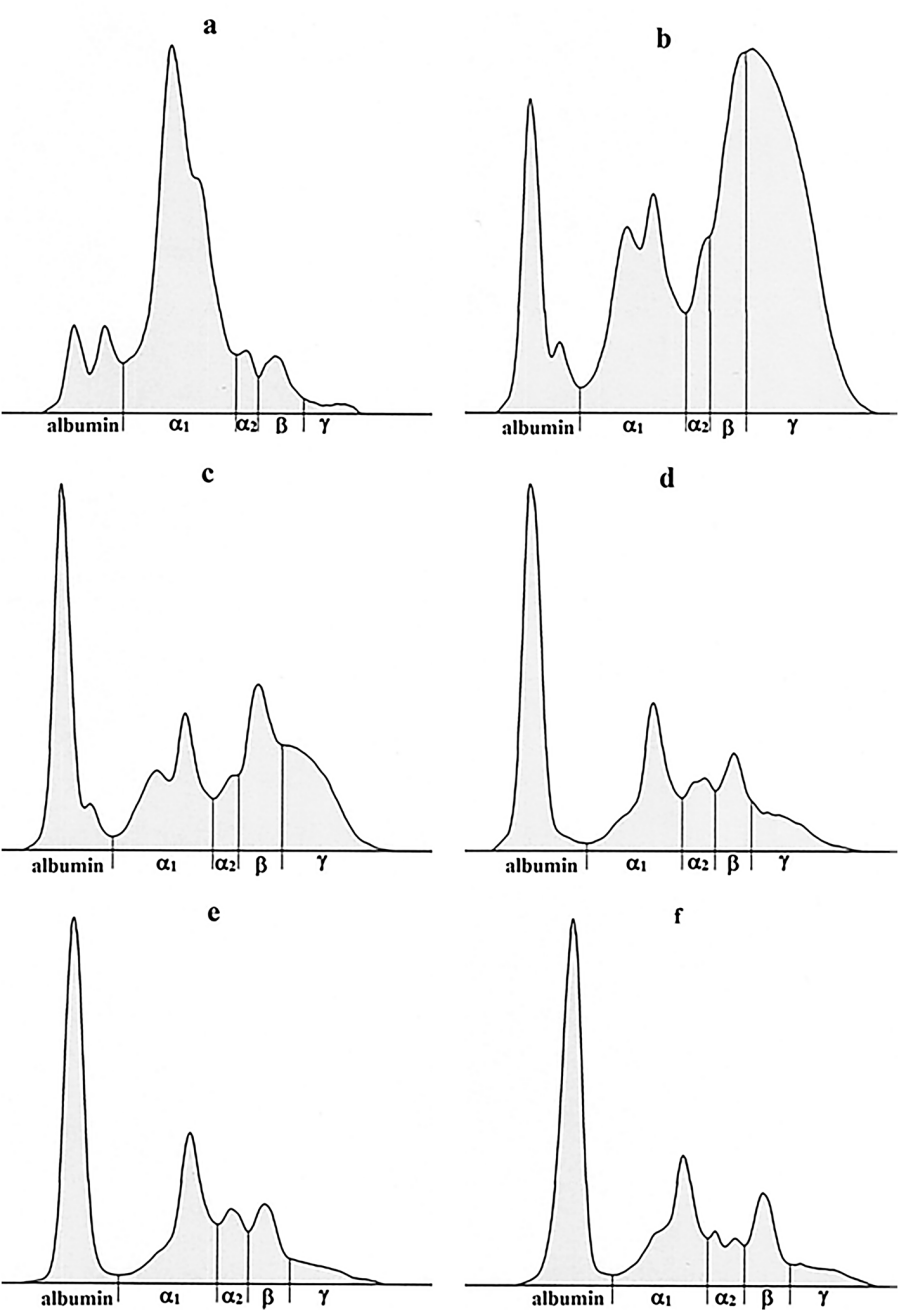
Representative electrophoretograms in a piglet showing the protein fractionation of serum proteins into five bands: albumin, α_1_-, α_2_-, β- and γ-globulins during the monitored period—(**a**) precolostral serum, (**b**) on day 2, (**c**) 1 week, (**d**) 2 weeks, (**e**) 4 weeks and (**f**) 6 weeks after birth.

Serum protein electrophoresis in piglets identified five distinct bands comprising albumin, α_1_-, α_2_-, β- and γ-globulins. The results of protein analyses are presented in Tables 1 and 2. Representative example of the electrophoretograms showing the protein fractionation during the evaluated postnatal period is presented in Figs. 1 and 2.

**Table 1:** Changes of the relative concentrations of serum protein fractions (%) and albumin/globulin ratios (A/G) in piglets during the evaluated period (mean ± SD).

Parameters		Blood sampling—age of piglets						*P* value
		0	2d	1w	2w	4w	6w	
Albumin	x	16.74^a^	16.12^a^	30.99^b^	49.67^c^	58.53^d^	55.74^d^	< 0.001
	± SD	3.06	3.06	5.23	7.12	3.39	4.59	
α_1_-Globulins	x	68.04^a^	24.67^b^	26.52^c^	21.34^d^	20.28^d^	21.85^d^	< 0.001
	± SD	4.24	3.51	3.76	2.65	1.85	1.85	
α_2_-Globulins	x	4.32^a^	5.54^b^	6.50^c^	5.99^b,c^	6.09^b,c^	5.42^b,d^	< 0.001
	± SD	1.02	1.31	1.15	1.08	1.30	1.17	
β-Globulins	x	8.48^a^	17.57^b^	15.26^b^	11.42^c^	9.53^a^	11.23^c^	< 0.001
	± SD	1.62	3.80	4.33	2.14	1.52	1.31	
γ-Globulins	x	2.42^a^	36.11^b^	20.53^b^	11.38^c^	5.56^d^	5.63^d^	< 0.001
	± SD	1.07	6.12	5.93	4.25	1.33	1.47	
A/G	x	0.20^a^	0.19^a^	0.46^b^	1.03^c^	1.43^d^	1.28^d^	< 0.001
	± SD	0.05	0.04	0.11	0.26	0.19	0.24	

*0* after birth, *d* days, *w* weeks, *P value* results of the analysis of variance (Friedman test); ^a,b,c,d^Different superscripts indicate the significance of the differences in means (*P* < 0.05).

**Table 2:** Changes of the absolute concentrations of serum total proteins (TP) and protein fractions (g/l) in piglets during the evaluated period (mean ± SD).

Parameters		Blood sampling—age of piglets						*P* value
		0	2 d	1 w	2 w	4 w	6 w	
TP	x	26.1^a^	56.8^b^	54.1^c^	49.1^d^	49.6^d^	47.8^e^	< 0.001
	± SD	2.3	5.5	6.1	4.3	3.8	2.7	
Albumin	x	4.4^a^	9.1^b^	16.9^c^	24.5^d^	29.1^e^	26.6f.	< 0.001
	± SD	1.0	1.5	3.8	4.0	2.9	2.4	
α_1_-globulins	x	17.8^a^	13.9^b^	14.2^b^	10.4^c^	10.0^c^	10.4^c^	< 0.001
	± SD	2.0	1.5	1.5	1.2	1.0	1.1	
α_2_-Globulins	x	1.1^a^	3.1^b^	3.5^b^	3.0^c^	3.0^b,c^	2.7^c^	< 0.001
	± SD	0.3	0.8	0.6	0.6	0.7	0.5	
β-Globulins	x	2.2^a^	10.0^b^	8.5^c^	5.7^d^	4.8^d^	5.4^d^	< 0.001
	± SD	0.5	2.4	3.1	1.4	1.0	0.7	
γ-Globulins	x	0.6^a^	20.7^b^	11.1^c^	5.6^d^	2.7^e^	2.7^e^	< 0.001
	± SD	0.3	4.8	3.3	2.0	0.6	0.8	

*0* after birth, *d* days, *w* weeks, *P value* results of the analysis of variance (Friedman test); ^a,b,c,d,e,f^Different superscripts indicate the significance of the differences in means (*P* < 0.05).

The analysis of the relative concentrations of all serum protein fractions showed significant changes during the evaluated period of postnatal development (Table 1; *P* < 0.001).

The mean value of albumin obtained immediately after birth (day 0), as well as 48 h after birth were low making up on average only 16.74% and 16.12% of total proteins, respectively and was among the protein fractions the second prominent fraction. Albumin values increased significantly approximately two-fold at the age of 1 week, followed with a further gradual significant increase of values in the next monitored period. The highest relative mean values of albumin were found 4 weeks after birth before weaning and its values decreased non significantly again after weaning. The relative concentrations of α_1_-globulins recorded were immediately after birth very high and the most prominent fraction with an average value of 68.04%. Subsequently, from day 2 after birth, a significant decrease (more than 40%) in the relative values of α_1_-globulins was observed. The lowest and non-significantly different mean values of this fraction were found in 2 to 6 week old piglets. Significant variations were found also in the relative concentrations of α_2_-globulins. They showed an opposite tendency of changes, a significant increase in values from 2nd day after birth. The highest relative mean values of α_2_-globulins were recorded in piglets between 1 and 4 weeks of age and after weaning the values decreased. The mean relative concentration of β-globulins was the lowest immediately after birth and the values were significantly the highest (about two times) 48 h after birth. From the age of 1 week, a gradual decrease in β-globulin values was observed up to the period before weaning and after weaning the values increased again. Gamma globulins represented the smallest proportion of protein fractions after birth with an average value of 2.42%. A significant increase of values was found 2 days after birth. The mean proportion of γ-globulins in total proteins was almost 40% and the values were this time the highest. From the 1st week of age, a significant gradual decrease in the proportion of γ-globulins was observed and the lowest and similar mean values as before colostrum intake were found before (4 weeks) and after weaning (6 weeks). The lowest A/G values were recorded immediately after birth and 48 h after birth. The mean A/G values increased significantly approximately two-fold in the age of 1 week with a further gradual significant increase until the week 4 after birth. After weaning the mean A/G values were non-significantly lower compared to the blood sampling before weaning.

The absolute concentrations of total serum proteins and all serum protein fractions showed significant changes during the evaluated period of postnatal development (Table 2; *P* < 0.001). The concentrations of TP were the lowest immediately after birth and increased markedly after the sucking. The mean value was significantly the highest 2 days after birth (more than two times higher). Subsequently, from the week 1 of age the mean TP concentrations started gradually significantly decrease until the end of the evaluated postnatal period with the lowest mean values found after weaning. Compared to globulins, the absolute concentrations of albumin immediately after birth were very low. In the following period, a significant gradual increase of its concentrations was found until the pre-weaning period (4th week), when the mean albumin concentration was the highest (approx. sixfold higher than the pre-colostral mean value). After weaning the mean albumin value was again significantly lower.

An opposite trend was found in the α_1_-globulins showing the significantly highest mean value immediately after birth and decreasing values until the age of two weeks. Significant alterations were observed also in the absolute concentrations of α_2_-globulins. Their concentrations increased significantly on day 2 and 1 week after birth and a subsequent significant decrease of values was recorded in the pre-weaning and post-weaning period. Similar tendency in the pre-weaning period was found in the changes of β-globulin concentrations. Their absolute concentrations increased significantly about 4.5-fold two days after birth and then started gradually to decrease until the 4st week of age. One week after weaning a slight non-significant increase of the values was observed. Immediately after birth, only minimal concentrations of γ-globulins were found in piglets. After sucking and colostrum intake the mean concentration of γ-globulins significantly increased more than 30-fold two days after birth. From the age of 1 week the values started markedly decrease until the age of 4 weeks and then remained at the same low levels also after weaning.

Electrophoresis (Fig. 1) showed in newborn piglets a double-peak in the albumin zone with two approximately similarly sized lower peaks (Fig. 2a). In two days old piglets was the albumin zone characterized by a more prominent higher peak in the anodal side and a minor peak on the cathodal side (Fig. 2b). In 1 week old piglets this peak appeared only as a shoulder after the main albumin fraction (Fig. 2c) and from the second week of life only a typical one peak of albumin was observed. The α-globulins migrated in α_1_- and α_2_-fractions and the α_1_-globulin fraction was the most prominent in newborn piglet's serum observable as a wide and high peak on the electrophoretogram (Fig. 2a). Its size decreased with advancing age from the second week of life the shape and size stayed relatively stable (Fig. 2d,e). The α_2_-globulin zone in newborn piglets was characterized by one peak, but after weaning it showed a separation in two small peaks (Fig. 2f). Serum protein electrophoresis identified in the β-globulin zone one overall distinct band, except of the period 48 h after the first sucking, when the β-globulins increased markedly and formed with the γ-globulins the β-γ fusion (Fig. 2b). The γ-globulin peak in neonatal piglets was very low (Fig. 2a), and a sharp increase 48 h after the first sucking was found forming with the β-globulin peak the aforementioned β-γ bridging (Fig. 2b). Its shape and size started gradually decrease from the 1st week of life (Fig. 2c–f). The γ-globulin peaks were during the pre-weaning and post-weaning period again low and clearly distinguishable from β-globulin zone.

## Discussion

The physiology of young animals is different from adults and may be markedly affected by growth, development, stressful conditions, as well as feeding, especially of colostrum or milk, which may have a bigger impact on the metabolism and health state in comparison with the adults^12^. It was found that the neonatal period in piglets is characterized by intensive changes in carbohydrate and fat metabolism^17,18^. In this study presented results showed important alterations also in the protein composition of blood serum in piglets during the postnatal development. The concentrations of total serum proteins in piglets immediately after birth were very low (around 25 g/l) and increased approximately two-fold two days after the first sucking. Bengtsson^19^ found in newborn piglets also low concentrations of total serum proteins and their values increased about two-fold after 3 days of sucking. Similarly, Dvorák^20^ and Martin et al.^10^ presented the lowest concentrations of total proteins in the newborn piglets before colostrum intake, but shortly after the ingestion of colostrum, the total proteins reached values of more than 70 g/l (approximately a three-fold increase). These higher concentrations decreased slightly until weaning, which is in agreement with our results.

The increase of total serum protein concentrations was accompanied by important qualitative, as well as quantitative changes in the protein fractions. At birth, the serum concentrations of albumin in piglets are relatively low, because its synthesis during the fetal development starts later when compared to other serum proteins, and due to the epitheliochorial type of the placenta the molecule transfer is not possible not only for immunoglobulins, but also for albumin^21,22^. According to Ingvarsson et al.^3^ and Martin et al.^10^, albumin represents only approximately 6–7% of total proteins in the serum of newborn piglets, which represents its significantly lower ratio compared to the results recorded in the presented work. The average albumin values obtained in newborn piglets by these authors were lower (3 g/l and 2.4 g/l) compared to the concentration recorded in our study. Slightly higher values were presented in the study conducted by Szymeczko et al.^23^, in which albumin constituted in average 14.2% of the total proteins. These results are similar to findings recorded in our study. The hepatic synthesis of albumin is markedly activated from the first day of postnatal life, which is reflected by rapid increase of serum concentrations^24^. Similarly, Bengtsson^19^ obtained in newborn piglets low concentrations of albumin, which was followed by a significant increase of values after sucking for 4 h. According to Page^25^, the hepatic synthesis of albumin increase about tenfold from birth to approximately 10 days of age, while Dvorák^20^ observed a gradual increase of albumin concentrations from birth until the beginning of the third month of age.

Albumin is one of the major proteins in the colostrum of pigs^26^. The concentration of albumin in the sow´s colostrum is high at farrowing and decrease rapidly during the first day of lactation^27^. The piglets may absorb albumin from the colostrum during the first hours of life, and its uptake by mucosal cells ceases 24–36 h after birth^28^. Thus, the increase of the concentrations of albumin in the piglets 48 h after the first sucking (from 4.4 to 9.1 g/l) may be partly the result of its absorption from the colostrum and partly by de novo synthesis by the piglets^3^. On the other hand, some authors stated that the intake of colostrum has no great influence on the increase of serum albumin concentrations in the piglets after birth and the increased synthesis of albumin in the liver of newborn suckled piglets is sufficient to account for its increased concentrations postnatally^20,25^.

On the electrophoretogram presented in the study the albumin zone in newborn piglets was characterized by two peaks. The shape and size of these peaks changed with advancing age, showing a more prominent, major and higher peak in the anodal side (moving faster) and a minor peak on the cathodal side of the albumin zone (moving slower), which probably represents the so called postalbumin fraction. This pattern of very low albumin concentrations in newborn piglets differs markedly from those obtained in other animal species and the presence of double albumin zone was previously not described in any other species. A minor postalbumin fraction was described only in equine serum as a shoulder on the cathodal side of the albumin peak^29^. The existence of serum albumin, prealbumin and postalbumin was previously suggested also in perinatal pigs by Lardinois and Page^30^, but their functions were not clearly described. Postalbumin could be a fetal-specific protein that disappear with advancing age^31^. Because up to now there is in the literature no exact explanation for the specific findings in albumin zone pattern in the first week of piglets life reported many years ago as well as in the presented study, further research would be helpful to clarify the biochemical and immunological properties of postalbumin in newborn piglets.

The predominant serum protein in newborn piglets before sucking is α_1_-acid glycoprotein, which represents approximately 50% of total serum proteins at birth, while in adult pigs decreases approximately 30-times and constitutes only 0.3% of blood proteins. Its concentrations in newborn piglets are around 12 mg/ml, whereas in adult pigs it presents only values about 0.25 mg/ml^14,32^. Similarly, Heegaard et al.^33^ showed higher serum concentrations of α_1_-glycoprotein in perinatal piglets compared to one month old ones. They found 2–5 days old piglets to have a mean serum concentration of α_1_-glycoprotein of 6.6 mg/ml, possibly reflecting a rapid decrease of its concentrations in the first few days after birth, while in 1 month old pigs a mean serum concentration of 1.1 mg/ml was found. According to Lampreave and Piñeiro^7^, the concentrations of α_1_-glycoprotein in this critical period may be an adaptive response to prepare the newborn pig for the extrauterine life. Furthermore, α_1_-glycoprotein was described as general acute phase protein in pigs^32,34^. The blood serum of newborn piglets is rich also in α_1_-antitrypsin and fetuin (may represent approximately 18% of total serum proteins at birth), which play important role by the inhibition of the effect of proteases absorbed by the gastrointestinal tract in the first days of life^7,13^. Although α_1_-fetoprotein is a major serum protein in fetal life, in contrast to other animal species, its concentrations in porcine fetuses and neonatal piglets are lower than those of fetuin and α_1_-antitrypsin^35,36^. The aforementioned proteins belong to the α_1_-globulin fraction and their high concentrations in newborn piglets might account for very high α_1_-globulin values observed in our study in piglets immediately after birth.

The protein pig major acute phase protein (pig-MAP) shows similarity with the proteins from the inter-α-trypsin inhibitor family^37,38^, therefore, it may also migrates into the α_1_-zone or α_1_-α_2_ interzone. In the first days after birth, a marked increase of serum pig-MAP concentrations was observed in piglets^10^, indicating that colostrum could be a source of this protein. Furthermore, the activated synthesis of pig-MAP by the liver may be another cause of its increased serum concentrations during the postnatal development. On the other hand, pig-MAP belong to acute phase proteins, thus, the rapid increase of its concentrations may suggest some kind of acute phase response to the rapid growth and various environmental factors^39^. According to Piñeiro et al.^40^, pig-MAP is considered an important stress marker, which concentrations increase in stressful conditions or due to changes in the composition of diet and in the pattern of feed administration.

Szymeczko et al.^23^ stated also that in newborn piglets α-globulins are the dominant fractions, but from these α_2_-globulins account for a larger proportion (about 50% of total proteins). In contrast to these data, our results suggest that α_1_-globulins compose the largest fraction of serum proteins and by visual estimation represent the most marked zone on the electrophoretogram, observable as broad and high peak in newborn piglets. In context of the literature data, according to which α_1_-glycoprotein is the predominant protein in neonatal piglets, we assumed that the markedly high peak observable on the electrophoretogram is part of the α_1_-fraction.

The α_2_-globulin fraction includes some acute phase proteins such as haptoglobin, α_2_-macroglobulin, as well as ceruloplasmin^41^. From these, α_2_-macroglobulin, ceruloplasmin, haptoglobin, as well as lipoproteins, have been found in fetal pig serum at the end of fetal development and in neonatal piglets, but they are all minor components^42,43^. Martin et al.^10^ observed in piglets a marked increase of haptoglobin concentrations during the first days of life, while these values were more than two-fold higher than those recorded in 6-month old pigs. Piñeiro et al.^44^ recorded in growing pigs a trend of increasing haptoglobin concentrations with age reaching a maximum at 12 weeks of life, while the higher values observed in the nursery period might be due to stress caused by weaning, separation from the mother, regrouping, changes of environment, as well as diet. Pié et al.^45^ described also an acute phase response after weaning, which was associated with increased expression of proinflammatory cytokines at days 0–2 post weaning. On the other hand, in the study conducted by Perri et al.^46^, haptoglobin was not influenced by the age and weaning, but the values of pig-MAP were higher in 3 weeks old piglets than in those after weaning. Transferrin and complement are the main components of the β-globulin fraction^47^. The major function ascribed to transferrin is the transport of iron ions from the intestine and the reticuloendothelial system to all proliferating cells in the body^48^. According to Thoren-Tolling and Martinsson^49^, its concentrations at birth are low (approximately 1 mg/ml), which is consistent with the transitory iron deficiency state in newborn piglets. The increase of β-globulins observed in our study in piglets from the second day of life may in part reflect the aforementioned increase of serum transferrin concentrations. Furthermore, some immunoglobulins absorbed from the colostrum, mainly IgA and IgM^5,50^, may migrate into the beta-fraction and contribute to the increase of β-globulins in piglets 48 h after colostrum intake and their subsequent gradual decrease associated with the degradation of these immunoglobulins.

The very low γ-globulin fraction in piglets immediately after birth reflect the lack of immunoglobulins in their serum before sucking, indicating (similarly to newborn calves) the existence of a barrier between the sow and the fetus against large molecules, including maternal immunoglobulins. The placenta of sows isolates the fetus from the maternal plasma proteins^51^, and therefore the maternal immunoglobulins, some other physiologically active proteins and bioactive peptides with different functions must be absorbed from the colostrum due to the intestinal mucosa^52,53^. The newborn piglets are reliant on these immunoglobulins absorbed from colostrum for passive humoral immune protection until their own immune system has sufficiently matured to produce antibodies against foreign antigens^11^. A very small amount of immunoglobulins may be detected in the blood of newborn piglets as result of selective transport. Piglets are functionally undeveloped and are dependent on maternal immunoglobulins for immune protection at least during the period from birth until weaning^54,55^. Furthermore, trace quantities of immunoglobulins may be synthesized de novo, predominantly by the thymus, spleen, liver and bone marrow, which belong to the main lymphopoetic tissues during the fetal development^56^. After the first intake of colostrum the immunoglobulins (mainly IgG) rapidly enter the systemic blood circulation and their serum concentrations increase markedly^57,58^. This pattern was observable in our study in the γ-globulin fraction, manifested by significantly increased concentrations 48 h after the first sucking. Bengtsson^19^ stated also that sucking for 4 h is accompanied by a sharp increase of β- and γ-globulins, while Szymeczko et al.^23^ observed in the 12th h of life an over 100-fold increase of γ-globulins compared to the approximately 34-fold increase obtained in our study.

The analysis of the obtained results showed relatively high standard deviations, which suggests a wider range and greater variability of values in the evaluated piglets. This pattern might be influenced by a variety of factors, among which the amount of IgG available to individual piglets and consumed may be the most important. Bland and Rooke^59^ stated that the initial concentrations of IgG in colostrum are very variable even within sows in the same unit. Differences were found also between different parts of the udder, while caudal teats tend to have lower IgG concentrations compared with the cranial teats^60^. Other factors, which influence the intake of immunoglobulins by the piglet are related to its ability to compete successfully and suckle, the position of the piglet in the birth order, as well as to the intestinal permeability and efficiency of transmission of IgG from the lumen of the intestine to the bloodstream prior to the gut closure^11,61^. This substantial variability between piglets to transfer IgG from the gut to the bloodstream may be explained by different birth weight, prematurity and several genetic factors^62,63^.

From immunoglobulins, IgG is the most clinically important globulin for piglets during the first weeks of life. The immunoglobulins absorbed from the colostrum and then from milk provide in piglets the passive immunity, until the organism is capable to initiate their synthesis de novo. According to Rooke et al.^64^, this process in piglets is possible from the 7th day of life. Curtis and Bourne^65^ stated that the half-lives of IgG in the blood serum of piglets range from 6.5 to 22.5 days, and that any IgG production by young piglets in the first 2–3 weeks of life contributes little to serum IgG concentrations. In the study of Martin et al.^10^, the IgG concentrations decreased progressively due to catabolism until the age of 8 weeks, when they reached minimum values. According to Curtis and Bourne^5^, the serum concentrations of immunoglobulins after this period start to increase again, as the active synthesis of IgG surpasses its breakdown. In our study, the concentrations of γ-globulins decreased markedly from the 2nd day of life until the age of 4 weeks reaching very low values, and this finding remained in piglets also 1 week after weaning. Due to this status of the immune system, the weaning and early post-weaning period are the most critical periods in the life of piglets, when they are susceptible to many important diseases resulting in growth retardation, even death. Furthermore, many stress factors associated with this period, such as removal from the sow, dietary changes, adapting to a new environment, mixing of piglets from different litters intestine, may negatively affect the immunological responses of the organism and result in serious diseases^66^. The aforementioned alterations in the composition of serum proteins during the postnatal development resulted also in marked changes of A/G ratio, and were primarily caused by changing globulin pattern during the growth and development.

## Conclusions

The presented study significantly expands the current knowledge in the analysis of the serum protein fractions in piglets and showed marked differences in the composition of serum proteins in newborn piglets before colostrum intake and during their postnatal development including the period after weaning. The results suggest marked developmental alterations in the serum protein pattern along with the age. Among these alterations observed, the changes in albumin, α- and γ-globulin fractions are of note. The serum protein electrophoretic pattern of newborn piglets before the intake of colostrum were characterized by very low concentrations of total proteins, albumin and γ-globulins and very high proportion of α_1_-globulins. Forty-eight hours after the first sucking there was a significant increase of total proteins and γ-globulins and significant decrease of α_1_-globulins. The changes observed in further periods of postnatal development were progressive and were characterized by a gradual decrease of total proteins, β- and γ-globulins, and further increase of albumin concentrations until the period before weaning. The changes in protein profile after weaning as a critical period for piglets was characterized by a decrease of total protein and albumin fraction and increase of β-globulin fraction. No significant changes were found in γ-globulin values. The aforementioned changes in the serum protein pattern may be associated with physiological changes during rapid growth and development, intensive metabolic processes, as well as changes in feeding and stress due to weaning. The study also brings new knowledge in the field of description of typical features of electrophoretograms in piglets in the observed period of their life. Seeing that the serum protein electrophoretic pattern in newborn piglets is not completely understood, there is a need for further investigations to improve understanding of changes occurring in the serum protein pattern and the obtained specificities in piglets during postnatal development.

## Methods

### Ethics declarations

This study was based on the standard clinical examination and blood sample collection. The blood samples were collected as per standard sampling procedure used without any harm to the animals. All procedures with animals in the study were conducted in accordance with the ethical standards and relevant quidelines and regulations. The experimental protocols were approved by the institutional Ethical Committee of the University of Veterinary Medicine and Pharmacy in Košice on protection of animals used for scientific purposes (26/11/2019) and complied with the institutional requirements of the Code of Ethics for Scientists (Directive 74/2019/UVLF). The study was carried out in compliance with ARRIVE guidelines.

### Animals and sample collections

A total of 50 newborn crossbreed piglets Large White x Landrace of both sexes (21 males and 29 females) from five sows (first to third gestation) were included into the study. The study was performed in the university experimental facility. The clinically healthy sows were housed individually in straw bedded farrowing crates, fed twice daily a commercial diet, parturitions were watched and occurred naturally. Environmental temperature of the farrowing room ranged between 20 and 22 °C and the farrowing pens were equipped with the heating lamp for piglets. After birth the piglets had their umbilical cord cut, roughly dried and weighted. The average number of piglets born alive was 11 per sow. The mean body weight of piglets at birth was 1.66 ± 0.28 kg. They were housed together with their mother until weaning in standard farrowing pens with straw bedding and space warming using a heat lamp to 28–31 °C. Each pen had a non-lidded hopper and a nipple water to ensure ad libitum feeding and free water access. The weaning of piglets was done at the age of 35 days (5 weeks), when they were moved from the farrowing to the nursery unit within the same object without transport. Piglets were fed from the day 10 of their life until the end of the evaluated period with the commercial complete feed intended for piglets from day 10 after birth until the age of the 14th day after weaning (Weaning pellets PP+, DeHeus, Czech Republic). The main chemical composition of the pellets and additives in the diet are presented in Table 3. The health status of the animals was evaluated daily until the end of the study using routine clinical diagnostic procedures, and was oriented to the observation of general health state, feed intake, weight gain and behaviour. The piglets were during the whole monitored period in a good general health status without any obvious clinical signs of diseases. The first blood samples from piglets were collected immediately after birth before colostrum sucking (day 0). The piglets were subsequently returned to the nursing sows allowed to suckle. Further blood sample collections were performed on day 2 (2d), 7 (1w), 14 (2w), 28 (4w) after birth (1 week before weaning), and 7 days after weaning (6w). Piglets were weighted individually before each blood sample collection. The average body weights in the after birth subsequent monitored periods were 1.81 ± 0.32 kg (2 days), 2.86 ± 0.69 kg (1 week), 4.74 ± 1.35 kg (2 weeks), 9.46 ± 1.97 kg (4 weeks) and 15.4 ± 2.68 kg (6 weeks).

**Table 3:** Chemical composition of the used piglet pellets and additives in the feed (in 1 kg of dry matter).

Components of feed		Additives	
Crude protein (%)	17.4	Vitamin A (IU/kg)	16 000
Crude fibre (%)	4.5	Vitamin E (mg/kg)	160
Fat (%)	5.4	Vitamin D3 (IU/kg)	1 200
Ash (%)	4.7	Cu (as copper sulphate, mg/kg)	139.0
Lysine (%)	1.22	Zn (as zinc sulphate, mg/kg)	109.0
Methionine (%)	0.44	Fe (as ferrous sulphate, mg/kg)	99.0
Ca (%)	0.5	Mn (as manganese oxide, mg/kg)	40.0
P (%)	0.52	I (as calcium iodate, mg/kg)	1.0
Na (%)	0.25	Se (as sodium selenite, mgkg)	0.2
		Se (as selenomethionine, mgkg)	0.1

Blood samples of approximately 1 ml were taken from the *sinus ophtalmicus* into serum gel separator tubes without additives and anticoagulants (Sarstedt, Nümbrecht, Germany). Animals for the blood collection from orbital sinus were manually restrained in dorsal recumbency position placed on V-board table. The head of the piglets was in front of the edge of the table and both the front and the back legs were held such they were pointed toward posterior of the pigs. After letting the blood samples to coagulate at room temperature, sera were obtained by centrifugation at 4000×*g* for 15 min and then transferred into Eppendorf tubes. The aliquots of sera were kept frozen at − 20 °C for further laboratory analyses.

### Laboratory analyses

To evaluate the changes in the protein profile in piglets serum samples were analyzed for the concentrations of total proteins and main protein fractions. The concentrations of total serum proteins (TP, g/l) were measured on the automated biochemical analyzer Alizé (Lisabio, Poully en Auxois, France) according to the biuret method with commercially available diagnostic kits (Randox, Crumlin, United Kingdom). Serum protein electrophoresis was performed according to the application notes of the manufacturer on agarose gel using an automated electrophoresis system Hydrasys and commercial diagnostic kits Hydragel 7 Proteine for the separation of serum protein fractions (Sebia Corporate, Lisses, Evry Cedex, France). Serum protein fractions were separated by zone electrophoresis on a buffered agarose gel at pH 8.8. Ten microliters of each serum sample were applied to performed, numbered sample wells on the agarose gel. Control serum (Control Serum Human Normal, Sebia Corporate, France) was included in each run of samples. The electrophoretic migration was performed 15 min at 20 °C constantly at 10 W, 40 mA, and 240 V. After migration, the gels were stained in amidoblack staining solution and then destained with acidic solutions and dried completely. After separation and staining of the gels, the staining intensity of individual protein bands was quantified using a densitometer Epson Perfection V700 (Epson America Inc., California, USA) in conjunction with image analysis software Phoresis version 5.50 (Sebia Corporate, France)^8^. The following protein fractions were identified: albumin, α_1_-, α_2_-, β- and γ-globulins. They were expressed as relative values (%) according to the optical density and their absolute concentrations (g/l) were quantified from the TP concentrations. Albumin:globulin ratios (A/G) were calculated as well.

### Statistical analyses

Descriptive statistical procedures were performed to calculate the arithmetic means (x) and standard deviations (SD) for each evaluated variable and sample collection time. The distribution of data was evaluated using Kolmogorov–Smirnov Test for normality. Not all the evaluated parameters showed normal distribution. Therefore, the data were subjected to analysis of variance using a non-parametric Friedman test to assess the age-dependent changes during the monitored period. The significance of differences in values between the sample collections was evaluated by Tukey’s Multiple Comparisons post-test. Significance was considered at 5% probability level. All calculations were carried out by the GraphPad Prism V5.02 (GraphPad Software Inc., California, USA) computer program.

## Supplementary Information

Supplementary Information.

## Data Availability

The datasets generated and/or analysed during the current study are available from the corresponding author on reasonable request.
