# Supplementary material for: Serum protein electrophoretic pattern in piglets during the early postnatal period
Source: Sci Rep. 2021 Sep 2;11:17539. doi: 10.1038/s41598-021-96957-6 (PMC8413273; doi:10.1038/s41598-021-96957-6)
Supplement: Supplementary file 1 — Supplementary Information. [file 41598_2021_96957_MOESM1_ESM.pdf]

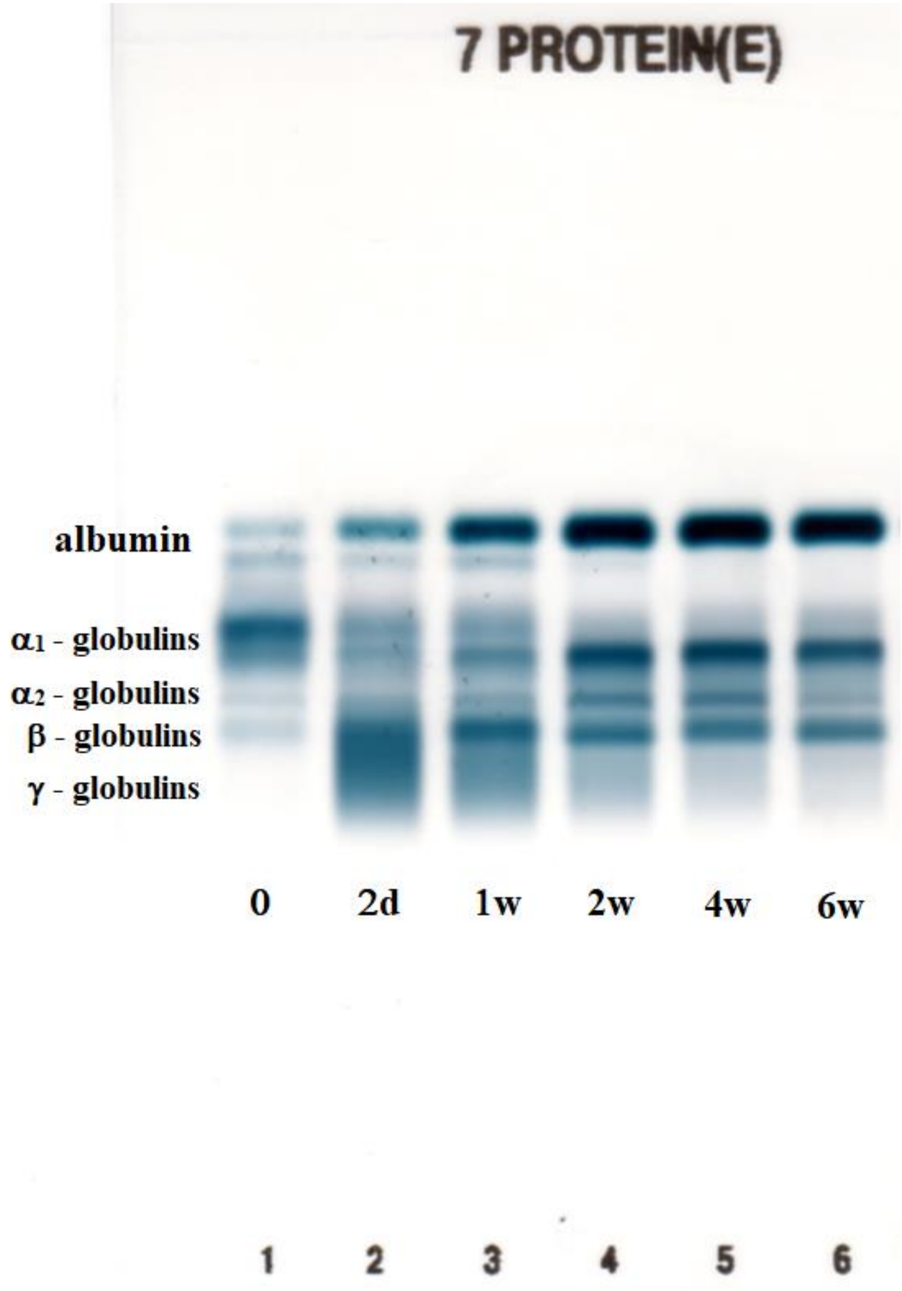

Supplementary figure S1. Electrophoretograms showing the protein fractions and changes in their proportion in a piglet during the early postnatal period – before colostrum inteke (**0**), on day 2 (**2d**), 1 week (**1w**), 2 weeks (**2w**), 4 weeks (**4w**) and 6 weeks (**6w**) after birth.
